# Modeling Forest Fire Occurrences Using Count-Data Mixed Models in Qiannan Autonomous Prefecture of Guizhou Province in China

**DOI:** 10.1371/journal.pone.0120621

**Published:** 2015-03-19

**Authors:** Yundan Xiao, Xiongqing Zhang, Ping Ji

**Affiliations:** 1 Research Institute of Forest Resource Information Techniques, Chinese Academy of Forestry, Beijing, P. R. China; 2 Research Institute of Forestry, Chinese Academy of Forestry, Beijing, P. R. China; Albert-Ludwigs-Universitat Freiburg, GERMANY

## Abstract

Forest fires can cause catastrophic damage on natural resources. In the meantime, it can also bring serious economic and social impacts. Meteorological factors play a critical role in establishing conditions favorable for a forest fire. Effective prediction of forest fire occurrences could prevent or minimize losses. This paper uses count data models to analyze fire occurrence data which is likely to be dispersed and frequently contain an excess of zero counts (no fire occurrence). Such data have commonly been analyzed using count data models such as a Poisson model, negative binomial model (NB), zero-inflated models, and hurdle models. Data we used in this paper is collected from Qiannan autonomous prefecture of Guizhou province in China. Using the fire occurrence data from January to April (spring fire season) for the years 1996 through 2007, we introduced random effects to the count data models. In this study, the results indicated that the prediction achieved through NB model provided a more compelling and credible inferential basis for fitting actual forest fire occurrence, and mixed-effects model performed better than corresponding fixed-effects model in forest fire forecasting. Besides, among all meteorological factors, we found that relative humidity and wind speed is highly correlated with fire occurrence.

## Introduction

Forest fire is one of the most dangerous natural hazards around the world. It does not only alter forest structure but also affect the forest carbon sink and the amount of greenhouse gases and aerosols. ‘Fire weather’ which refers to meteorological factors is conducive to forest fire, such as precipitation, air temperature, relative humidity, and wind speed [1, 2]. In Qiannan prefecture, above mentioned meteorological data are available which are automatically collected by meteorological stations, and such data can be collected in real time with low costs. Therefore, it is feasible and operational to predict forest fire based on the analysis of those meteorological factors.

**Fig 1:**
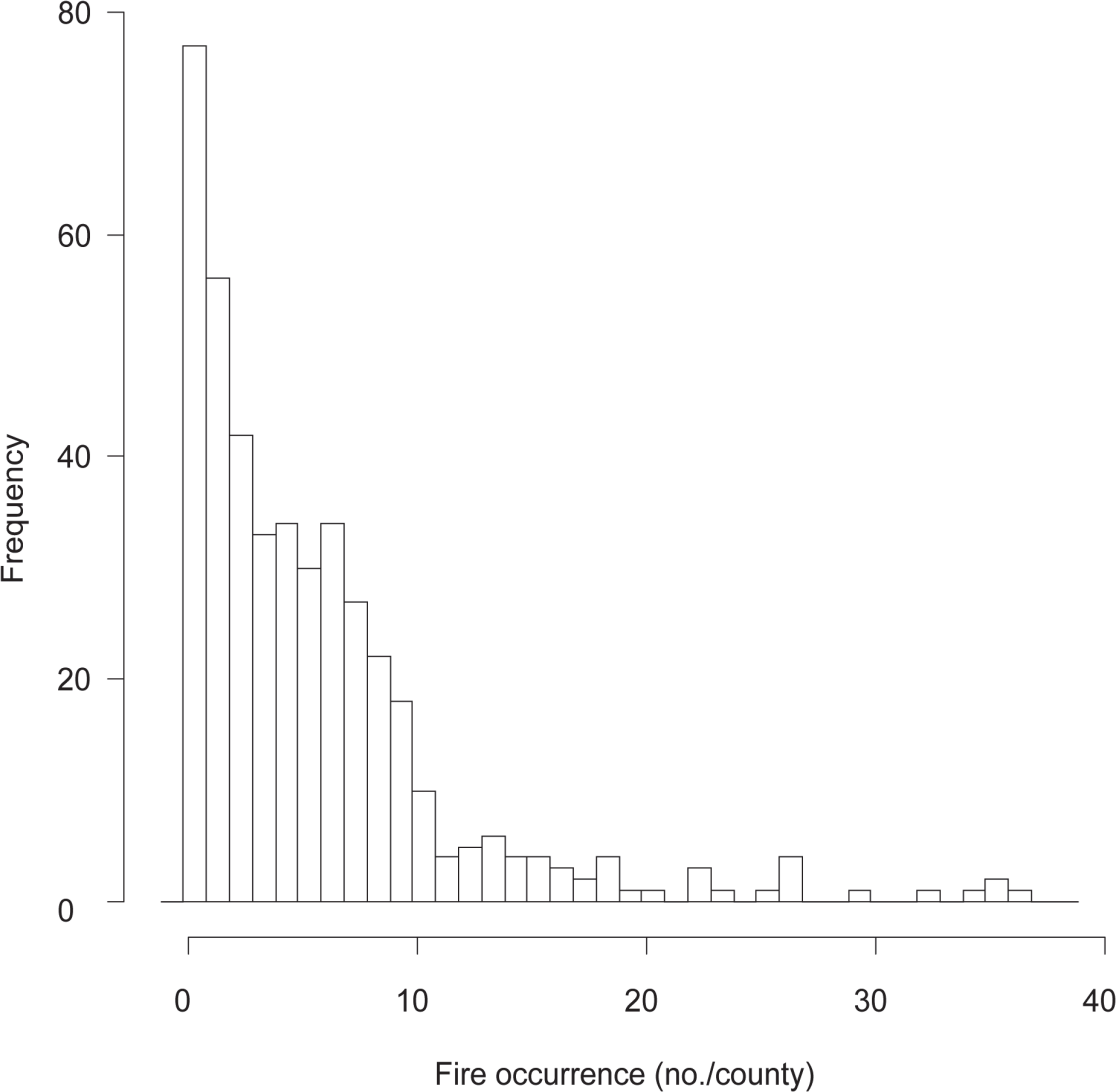
Histogram of forest fire occurrence data in the spring fire season from January to April between 1996 and 2007 in Qiannan autonomous prefecture of Guizhou province, China.

Because many counties in Qiannan have reported no occurrences of forest fires before (Fig. 1), fire data are bounded and characteristically exhibit varying degrees of dispersion and skewness in relation to the mean. Additionally, the data often contains an excess of zero counts. The least squares method implicitly presumes that the data are Gaussian distributed with constant variance, or at least satisfy the Gauss-Markov conditions. If this method is used for data processing with a large proportion of zero counts, the estimated results will be biased. Alternatively, if only forest fire observations are used for model development, forest fire occurrences will be overestimated.

To overcome this problem, some researchers have used a two-step approach to analyze the type of data used in the model [3]. With the two-step approach, the probability of zero occurrence is obtained from a logistic regression in the first step. Then the number of fires, given that some are known to occur, is described through a forest fire function fit by least squares in the second step. This method is notable in its recognition of an excess number of zeros as an aspect of the response distribution that is not well solved by standard nonlinear regression models and has been utilized with some modifications in other studies [4–6]. In recent years, there has been considerable interest in models for count data, such as species abundance [7], medical consultations [8], use of recreational facilities [9], stand mortality [10], and tree recruitment [11]. Few studies, however, have addressed this issue in forest fire prediction. Mandallaz and Ye [12] used Poisson models to predict forest fires in France, Italy, Portugal, and Switzerland and found that the prediction result obtained by using Poisson model is reliable. Wotton *et al*. [13] also used Poisson model to predict daily forest fire occurrence in Ontario. Xiao *et al*. [14] incorporated zero-inflated models and hurdle model techniques to forest fire equations and found that a zero-inflated negative binomial model performed better than other count-data models.

Forest fire occurrence is a complicated stochastic process influenced by several environmental factors, which often exist with complex interactions. It is impossible to capture all of the observed variability in forest fire data. Incorporation of random effects to take into account unforeseen and unexplainable variation is a common method. The evolution of the mixed-effects modeling methodology has provided a statistical method capable of explicitly modeling stochastic structure as a possible approach for solving this problem [15]. Although many forest models account for mixed effects, to our knowledge, there are no such reports for forest fire occurrence.

Due to the influences of geographical location, topography, climate and forest distribution, forest fires occur frequently in Qiannan Prefecture, Guizhou province, China, where the risk of forest fire is high from January to April [16, 17]. Although some researchers [13, 14] have used the count data models to develop forest fire occurrence models, in this manuscript, we introduced the mixed-effects to the count data models for accounting for the random effects of counties. To our knowledge, no study has used count-data mixed models to directly analyze the fire occurrences. The objective of this study was to develop and compare count-data models with and without random effects to predict forest fire occurrence using meteorological variables based on the forest fire occurrence and meteorological variables from January to April in Qiannan.

## Data

### Study sites

**Fig 2:**
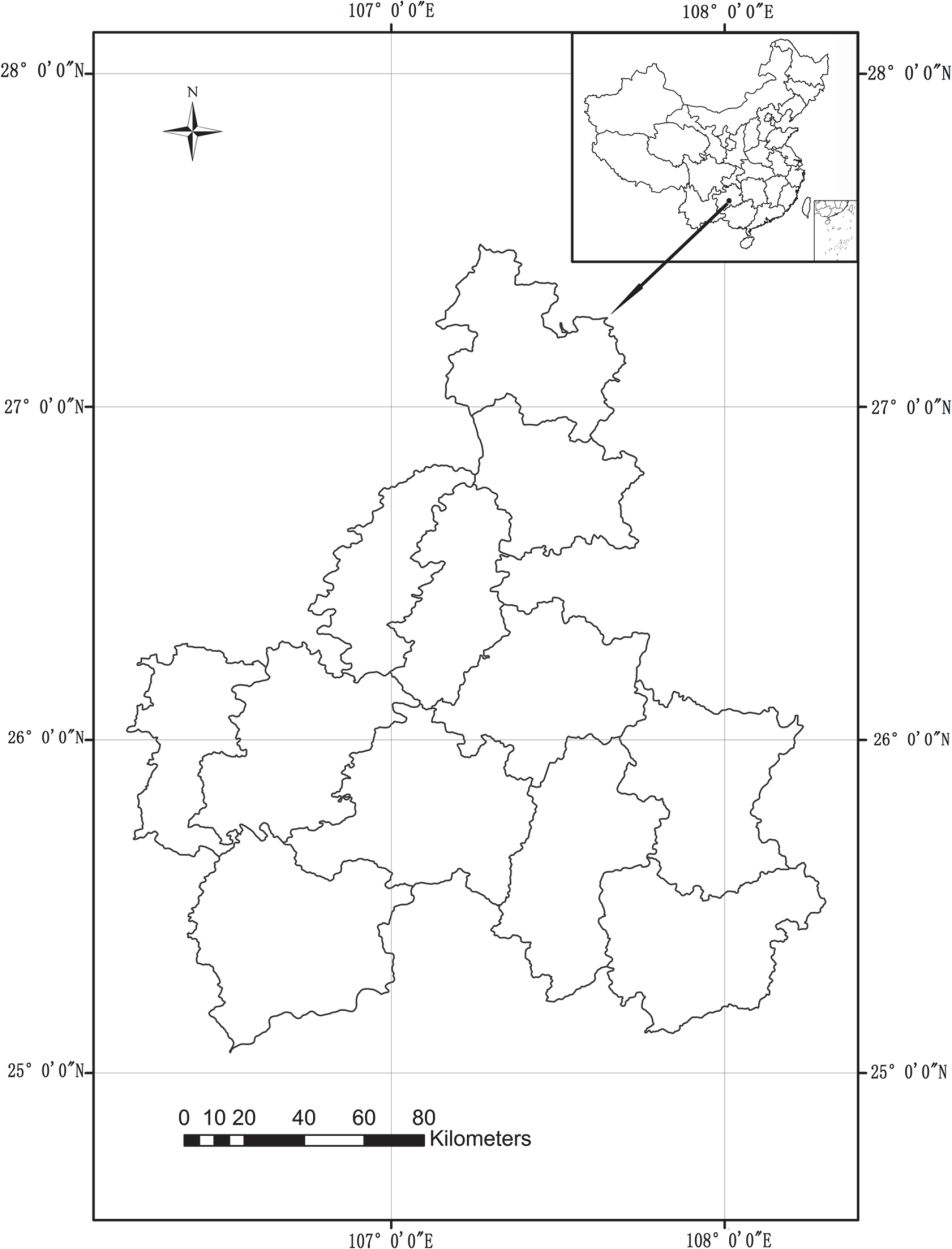
Location of Qiannan autonomous prefecture in Guizhou province, China.

Forest fire data were collected by the Forestry Bureau of Qiannan autonomous prefecture. Qiannan is located in south-central Guizhou province (Fig. 2), with a total area of 26, 200 km^2^, geographic latitude 25°04'~ 27°29', and longitude 106°21 '~ 108°18'. There are 12 counties in Qiannan: Weng'an, Fuquan, Guiding, Changshun, Huishui, Duyun, Pingtang, Dushan, Sandu, Luodian and Libo. Qiannan presents a terrain in which the northwest is higher than the southeast. With the vast percentage of its land comprised of mountains, Qiannan is considered as forest fire risk zone in Guizhou province. The Qiannan autonomous prefecture is in the subtropical monsoon climate zone. The average annual temperature ranges from 13.6 ~ 19.6°C, increasing gradually from north to south and from west to east. The annual average precipitation is 1100 ~ 1400 mm, and the annual average relative humidity is 80 to 83%. Within the year, the weather in spring is hot and dry, resulting in spring drought in Qiannan prefecture [16].

### Meteorological variables

The meteorological data from January to April between 1996 and 2007 used in this study were collected from 12 counties’ weather stations in Qiannan autonomous prefecture. The following meteorological factors were commonly available: monthly maximum temperature (*Tmax*), monthly mean temperature (*T*), monthly mean relative humidity (*H*), monthly mean wind speed (*S*), monthly maximum wind speed (*Smax*), monthly precipitation (*P*) and monthly evaporation (*E*). The statistics of these meteorological variables are listed in Table 1.

**Table 1:** Meteorological variables during the spring fire season in Qiannan autonomous prefecture from 1996–2007.

Meteorological Variables	Min	Max	Mean	SD.
Maximum temperature per month (°C)	10.9	23.5	24.51	4.91
Mean temperature per month (°C)	3.7	31.7	14.41	5.32
Mean relative humidity per month (%)	19	133.3	78.31	8.20
Maximum wind speed per month (m.s^-1^)	1	13.1	6.13	1.87
Mean wind speed per month (m.s^-1^)	0.1	3.9	1.56	0.63
Precipitation per month (mm)	0.1	130.8	35.64	23.18
Evaporation per month (mm)	17.1	147.1	64.71	25.83

## Method

Count data models are a subset of discrete-response regression models and aim to explain the number of occurrences or counts of an event. Such models have been applied in many situations. In this study, a Poisson model, negative binomial model, zero-inflated models, and hurdle models were used for modeling the relationship between forest occurrences and meteorological variables. In this study, these climate variables used to predict fire occurrence, including monthly mean temperature (*T*), monthly maximum temperature (*Tmax*), monthly mean relative humidity (*H*), monthly mean wind speed (*S*), monthly maximum wind speed (*Smax*), monthly precipitation (*P*) and monthly evaporation (*E*).

### Poisson model

Poisson model is the simplest model for count data, and the probability mass function (PMF) for a Poisson is characterized as follows:

$$P(Y=y)=\frac{e^{-\lambda }\lambda^{y}}{y!}$$ (1)

where *y* refers to random variable of count response, *y*_=_ 0, 1, 2, and *λ* > 0. A Poisson regression model is obtained by relating the mean *λ* to a vector of independent variables *X*, by *λ* = Exp(*Xβ*), where *β* is a vector of regression coefficients to be estimated. A characteristic of the Poisson probability function (Eq. 1) is that the mean and the variance are equal, that is, Var[*Y* | *X*] = E[*Y* | *X*] = *λ*. When data do not fit the Poisson distribution, it is typically resulted from overdispersion, meaning that the data’s variance exceeds the mean value.

### Negative binomial model

A negative binomial model (NB) can be considered a generalization of the Poisson model and addresses the issue of overdispersion by including a dispersion parameter to accommodate the unobserved heterogeneity in the count data [18]. The PMF for the negative binomial is given as follows:

$$P\left(Y=y\right)=\frac{\Gamma (y+\theta^{-1})}{\Gamma (y+1)\Gamma (\theta^{-1})}{\left(\frac{\theta^{-1}}{\theta^{-1}+\lambda }\right)}^{\theta^{-1}}{\left(\frac{\lambda }{\theta^{-1}+\lambda }\right)}^{y}$$ (2)

where *θ* represents the dispersion parameter. The mean is *λ*, as in the Poisson model, but the variance is *λ + θλ* ^2^, thus allowing the variance to exceed *λ*. The NB model is obtained by relating the mean *λ* to a vector of independent variables: *λ* = Exp(*Xβ*).

### Zero-inflated models

In zero-inflated models, two regression equations are created: one predicting whether the count occurs and the other predicting differences in the occurrence of the count [19]. Supposing that a discrete variable *Y* (number of fire occurrences) follows a zero-inflated distribution, the PMF for a zero-inflated distribution is given by

$$P\left(Y=y\right)=\{\begin{array}{c} p+(1-p)f(0)\\ \left(1-p)f(y\right) \end{array}\begin{array}{c} y=0\\ y>0 \end{array}$$ (3)

where *p* is the probability of belonging to the point mass at zero and (1-*p*) is the probability of belonging to the count component. Logistic regression is commonly used to fit the point mass component. In this study the count component corresponds to the probability mass function of a Poisson distribution (zero-inflated Poisson model, ZIP) or NB distribution (zero-inflated negative binomial model, ZINB).

### Hurdle models

Hurdle models, originally proposed by Mullahy [20], are the other mixture models. They consider the count outcome to be generated by two different statistical processes: a binomial distribution determining whether a count outcome is zero or nonzero and a truncated-at-zero distribution for count data governing all positive counts conditional on the count component. However, they are slightly different from zero-inflated models with all zero counts from two different sources and assume that zero counts might come from a single statistical process [21]. The PMF for a Hurdle model is given as follows:

$$P\left(Y=y\right)=\{\begin{array}{c} p\\ \left(1-p\right)\frac{f(y)}{1-f(0)} \end{array}\begin{array}{c} y=0\\ y>0 \end{array}$$ (4)

where *p* is the probability of a zero count and (1-*p*) is the probability of overcoming the hurdle. Similar to the zero-inflated models, a logistic function is often used to model the point mass at zero. As for the count component, it also corresponds to the probability mass function of a Poisson distribution (Hurdle Poisson model, HP) or NB distribution (Hurdle negative binomial model, HNB).

In the study, a county-level random-effect parameter was added to the intercept for the Poisson model and NB model. The random-effect parameter was defined as follows: *u*_1_~N(0, *v*_1_). Two random-effect parameters were added to the ZIP model, HP model, ZINB model and HNB model. The random-effect parameters were defined as *u*_1_, *u*_2_ ~N([0, 0], [*v*_1_, 0, *v*_2_]). The unstructured covariance structure [22] was used to describe the variance-covariance structure of the random effects. Estimation of parameters was implemented with the SAS/STAT NLMIXED procedure.

### Model selection and goodness-of-fit

A Poisson fixed-effects model, NB fixed-effects model, zero-inflated fixed-effects models, Hurdle fixed-effects models and corresponding mixed-effect models calibrated with the same data set can be compared through the Akaike information criterion (AIC) and Bayesian information criterion (BIC):

$$\text{AIC}=-2L(\hat{\varphi },y)+2k$$ (5)

$$\text{BIC}=-2L(\hat{\varphi },y)+k\ln(n)$$ (6)

Smaller values of the AIC and BIC indicate that a model is better. Both the AIC and BIC rely on a penalized maximum log-likelihood value. As the penalty is based on the number of model parameters, the criteria ensure the best trade-off between the goodness of fit and the number of parameters. The penalty is more influential in the BIC, making this criterion more conservative than the AIC [6].

Because both the AIC and BIC are relative statistics, they do not ensure that the fit of the “best” model is good. Hence, diagnostic plots, which plot the differences between predicted and observed probabilities against count class *j*, were used to detect any predictive bias and assess goodness-of-fit [6, 23]. The difference, *d*_*j*_, between predicted probabilities and observed probabilities was computed as

$$d_{j}=\sum_{i=1}^{n}\left(\frac{P\left(y_{i}=j\right)}{n}\right)-\left(\frac{\#y_{i}=j}{n}\right)$$ (7)

where # represents the frequency of observations *y*_*i*_ in count class *j*, *n* represents the number of count classes, and *P*(*y = j*) is the predicted probability that an observation belongs to count class *j*.

## Results

According to T-test, some variables with *p*-values > 0.1 were removed from the models. In this study, one random-effect parameter was added to the intercept of the Poisson model and negative binomial model, and two random-effect parameters were added to zero-inflated models and hurdle models. To get convergent results for the zero-inflated mixed models and hurdle mixed models, we found that one random parameter was in the intercept of the count model and the other was in the evaporation variable of the zero model.

Among 12 models, the majority of variables were significant at the 0.05 level. In the count model, relative humidity was significantly negatively correlated with forest fire occurrence (*P*<0.05). In contrast, mean maximum wind speed was significantly positively correlated with fire occurrence (*P*<0.05, Table 2). In addition, fire occurrence was negatively correlated with evaporation in the study (*P*<0.05, Table 2).

**Table 2:** Parameter estimations and fit statistics for twelve models.

Parameter	Poisson-fixed	Poisson-mixed	NB-fixed	NB-mixed	ZIP-fixed	ZIP-mixed	ZINB-fixed	ZINB-mixed	HP-fixed	HP-mixed	HNB-fixed	HNB-mixed
Count model												
Intercept	2.78**	2.06**	2.69**	1.42**	2.72**	1.77**	2.75**	1.64**	2.73**	1.77**	2.71**	1.62**
*H*	-0.02**	-0.008**	-0.01**	-	-0.01**	-	-0.01**	-	-0.01**	-	-0.01**	-
*Smax*	0.07**	0.09**	0.08**	0.09**	0.06**	0.08**	0.08**	0.08**	0.06**	0.08**	0.09**	0.09**
*E*	-0.006**	-0.006**	-0.006**	-0.005**	-0.005**	-0.007**	-0.007**	-0.007**	-0.007**	-0.007**	-0.008**	-0.008**
*v*_*1*_	-	0.28**	-	0.23**	-	0.21**	-	0.19**	-	0.21**	-	0.17*
Zero model												
Intercept	-	-	-	-	-0.94**	-0.88**	-	-	-0.96**	-0.91**	-	-
*E*	-	-	-	-	-0.01*	-0.01*	-0.04**	-0.05**	-0.009*	-0.01*	-0.02**	-0.02**
*θ*	-	-	1.03**	0.99**	-	-	0.80**	0.76**	-	-	0.86**	0.84**
*v*_2_	-	-	-	-	-	-0.008**	-	0.02*	-	0.007**	-	0.008**
AIC	3613.4	3502.1	2370.1	2368.9	3128.8	3081.6	2370.7	2369.8	3128.5	3081.7	2372.5	2371.7
BIC	3629.7	3504.5	2390.4	2371.3	3153.2	3085.0	2395.1	2373.2	3152.9	3085.1	2396.9	2375.1

Note: ** significant at 0.05 level,

* significant at 0.1 level.

In the study, we also found that the AIC and BIC of NB mixture models (NB, ZINB, HNB) were much smaller than those of Poisson mixture models (Poisson, ZIP, HP) (Table 2). The AIC value of the Poisson fixed-effects model was 52.45% larger than that of the NB fixed-effects model, and the BIC was 51.84% larger.

**Fig 3:**
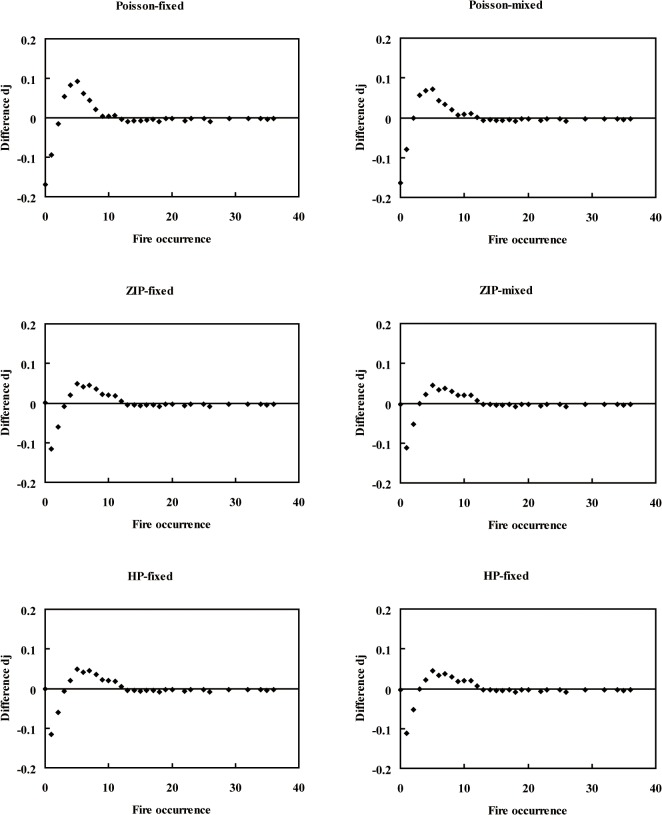
Diagnostic plots for the Poisson mixture fixed-effects models and mixed-effects models. d_j_ is the difference between the predicted probability and the observed probability, as shown in Equation (7).

**Fig 4:**
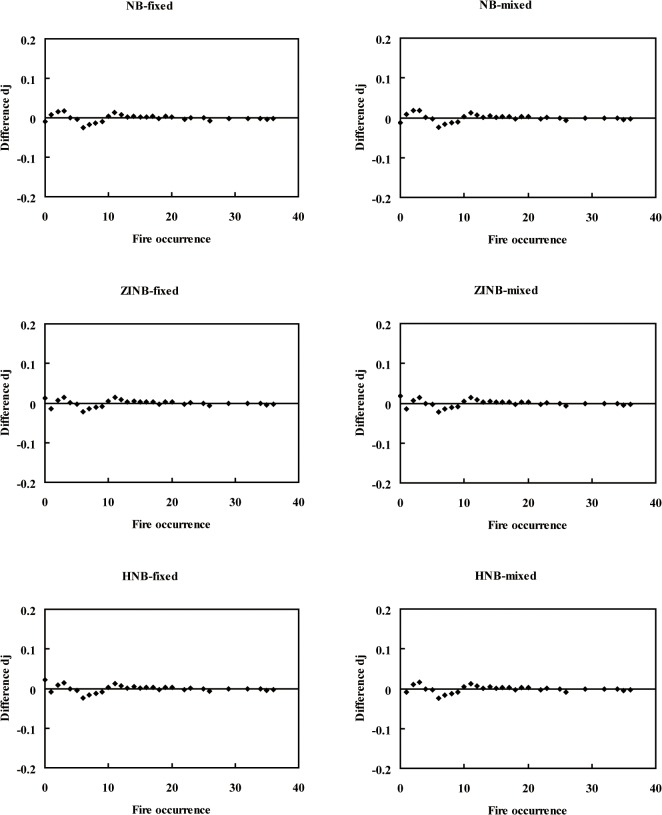
Diagnostic plots for the NB mixture fixed-effects models and mixed-effects models. d_j_ is the difference between the predicted probability and the observed probability, as shown in Equation (7).

In addition, mixed-effects models were used in the study. For the zero-inflated models and hurdle models, the parameter *u*_1_ was added to the intercept of the count model and *u*_2_ was added to the variable *E* of the zero model. We found that the Poisson fixed-effects model and Poisson mixed-effects model highly underestimated the zero-class counts (Fig. 3), and the remaining 10 models exactly estimated the zero occurrence counts (Fig. 4). In this study, the random effects were significant at the 0.05 level (Table 2). The difference *d*_*j*_ of the mixed-effects models was smaller than that of the fixed-effects models, and the NB mixed-effects model was smaller than the other mixed-effects models (Figs. 3, 4).

## Discussion

Meteorological factors are important factors for conducting forest fire. Relative humidity has an important impact on forest fires [24]. Long days of low humidity in a month tend to dry out all forest fuels to a dangerous level. Even a few days of low humidity can increase the risks of brush and forest fires. During fire suppression, the cycle of humidity can cause fires to burn with more intensity during the heat of the month [25]. That is, low relative humidity causes dry combustible materials and hence a high probability of forest fire [26]. In the study, the relation of relative humidity and forest fire occurrence was negative (Table 2), which is consistent with previous studies [27, 28]. Wind is the other meteorological factor that has a strong influence on forest fire occurrence, which may cause fuel drying as well as boost fire spread [29]. The effect of mean maximum wind speed on fire occurrence was positive (Table 2). Large forest fires in the counties of the Qiannan autonomous prefecture occur during the long spring fire season, which is characterized by dry fuels and strong winds [30]. High evaporation may contribute to an increased number of fire danger days in a month [25]. However, the relation of evaporation and fire occurrence was negative (Table 2). This may be explained that evaporation depends on a complex way with three major factors of temperature, humidity, and wind; the influence of any one of which may be offset by a pronounced change in either or both of the other two [31]. In a warmer climate, it is likely that overall fire occurrence would be higher as a result of increased temperature [32]. However, we found that the relationship between fire occurrence and temperature was not significant in this study.

Although the Poisson model is the simplest count data model, it is highly restrictive, as the variance of the outcome is assumed to equal its expectation. Count data sets always exhibit overdispersion. The NB distribution offers a dispersion parameter that well explains the overdispersion of positive count data [18]. Therefore, NB mixture models performed better than Poisson mixture models. We also found that the NB model was slightly better than the ZINB model and HNB model. The ZINB model and HNB model performed similarly in modeling fire occurrence, which was consistent with a previous study [11] finding that the ZINB model and HNB model have a similar advantage in modelling overdispersion data. The HNB model and ZINB model both furnish a composite predictor that does not enjoy the property that the mean and the variance are equal. Therefore, in this sense, the NB model, which allows for unobserved heterogeneity about a fire occurrence function, appears preferable to models that assign further structure to the mean [33].

Hall [7] added a random effect to account for within-subject dependence in the Poisson state of a ZIP model. However, fitting a zero-inflated random effect model is more complex than fitting a hurdle random effect model [34]. Li *et al*. [35] incorporated random effects into count data models to analyze tree ingrowth and found that it improved the model performance. Overall, the count-data fixed-effects models were shown to be improved by the inclusion of random effects (Table 2, Figs. 3, 4) as this accounted for most of the variation between data sources as well as county-to-county variability. The results indicated high county-to-county variability, which was unable to be explained by the fixed-effects models. Induced causes of county-to-county variability, such as topography, environment, and other unforeseen factors, cannot be defined and explained well.

It should also be noted that prediction of forest fire occurrence is a complex issue concerning weather, tree species, geography conditions and human activities. Non- meteorological factors may also play a considerable role in fire occurrence. For instance, fire spread may be influenced by topography, forest vegetation (distribution of fuel), and ignition rates by humans [30, 36]. Among the non-climatic factors, human activities in particular raise the probability of fire occurrence. These include human demographic patterns and activities, especially land use and fire management [37, 38]. Humans can also indirectly promote or restrain fires, e.g., by modifying landscape patterns, forest composition or fuel amounts. Substantial changes in fire frequency have also been found to be linked to changes in human population densities [39, 40]. In addition, high mountains and deep valleys are typical in the Qiannan forest areas, which favor severe or high-intensity fires and make fire suppression difficult or hinder fire suppression. We believe that if the non-climatic factors had been included in the analysis, the study would be improved and yield more accurate predictions.

## Conclusions

In this study, we have reviewed six count data models for forecasting forest fire occurrence in Qiannan prefecture. When the data is dispersed, the NB model, zero inflated model and hurdle model might give a more satisfactory fit to the data. Moreover, these models with mixed-effects were more appropriate when considering the random effects among counties. Based on the model comparisons, the NB mixed-effects model performed better than the other eleven models (including ZINB mixed-effects model and HNB mixed-effects model) in modeling fire occurrence in the study. We also found that the relative humidity was negatively correlated with fire occurrence. By contrast, maximum wind speed was positively correlated with fire occurrence. However, we found that the relationship between fire occurrence and temperature was not significant in this study.
